# Prognostic Autophagy-Related Model Revealed by Integrating Single-Cell RNA Sequencing Data and Bulk Gene Profiles in Gastric Cancer

**DOI:** 10.3389/fcell.2021.729485

**Published:** 2022-01-10

**Authors:** Tianying Tong, Jie Zhang, Xiaoqiang Zhu, Pingping Hui, Zhimin Wang, Qiong Wu, Jiayin Tang, Haoyan Chen, Xianglong Tian

**Affiliations:** ^1^ State Key Laboratory for Oncogenes and Related Genes, Division of Gastroenterology and Hepatology, Key Laboratory of Gastroenterology and Hepatology, Ministry of Health, Shanghai Institute of Digestive Disease, Renji Hospital, School of Medicine, Shanghai JiaoTong University, Shanghai, China; ^2^ Department of Clinical Laboratory, Renji Hospital, School of Medicine, Shanghai Jiao Tong University, Shanghai, China; ^3^ School of Biomedical Sciences, Li Ka Shing Faculty of Medicine, The University of Hong Kong, Hong Kong SAR, China; ^4^ Department of Gastroenterology, Tongren Hospital, Shanghai Jiao Tong University School of Medicine, Shanghai, China; ^5^ Department of Emergency, Luwan Branch of Ruijin Hospital, Shanghai Jiao Tong University School of Medicine, Shanghai, China; ^6^ Department of Gastrointestinal Surgery, Renji Hospital, School of Medicine, Shanghai Jiao Tong University, Shanghai, China

**Keywords:** gastric cancer, autophagy, single-cell RNA sequence, immune checkpoint, immunotherapy

## Abstract

Autophagy has been associated with tumor progression, prognosis, and treatment response. However, an autophagy-related model and their clinical significance have not yet been fully elucidated. In the present study, through the integrative analysis of bulk RNA sequencing and single-cell RNA sequencing, an autophagy-related risk model was identified. The model was capable of distinguishing the worse prognosis of patients with gastric cancer (GC), which was validated in TCGA and two independent Gene Expression Omnibus cohorts utilizing the survival analysis, and was also independent of other clinical covariates evaluated by multivariable Cox regression. The clinical value of this model was further assessed using a receiver operating characteristic (ROC) and nomogram analysis. Investigation of single-cell RNA sequencing uncovered that this model might act as an indicator of the dysfunctional characteristics of T cells in the high-risk group. Moreover, the high-risk group exhibited the lower expression of immune checkpoint markers (*PDCD1* and *CTLA4*) than the low-risk group, which indicated the potential predictive power to the current immunotherapy response in patients with GC. In conclusion, this autophagy-associated risk model may be a useful tool for prognostic evaluation and will facilitate the potential application of this model as an indicator of the predictive immune checkpoint biomarkers.

## Introduction

Gastric cancer (GC) is the most common cause of cancer-related morbidity and mortality worldwide (Bray et al., 2018; Smith et al., 2018). Despite the rapid advances of modern aggressive and comprehensive treatments, the 5-y survival rate of GC remains low (Ajani et al., 2017; Liu et al., 2020). Owing to the atypical clinical symptoms of GC at an early stage, most patients were diagnosed at the advanced stage and missed the chance of surgery therapy. Thus, an investigating predictive model for early diagnosis is essential to the improvement of the long-term survival rate and quality of life among patients with GC.

Autophagy, which is tightly regulated by a series of autophagy-related genes (ATGs), is an essential intracellular homeostatic process involved in the progression of cancer (Guo et al., 2013; Amaravadi et al., 2019) and is tightly linked to the regulation of pathways involved in the initiation and progression of cancer. Recent studies have demonstrated that autophagy-associated molecules show promise for the treatment of GC and autophagy-related inhibitors may enhance the therapeutic efficacy of immune checkpoint inhibitor in GC (Cao et al., 2019; Wang et al., 2019). However, the prognostic value of autophagy-related biomarkers in GC remains to be clarified unequivocally.

In current years, numerous studies have begun to uncover the crucial role of autophagy in the differentiation and function of the immune cells (Clarke and Simon 2019; Xia et al., 2021). Prior efforts to describe the tumor microenvironment of GC using bulk transcriptomic sequencing have enhanced our understanding of the association between the immune system and clinical outcome, but there exist some limitations in uncovering the complexity of cellular composition and the underlying mechanism involved in tumor initiation and progression (Frishberg et al., 2019). The advances of single-cell RNA sequencing (scRNA-seq) have revolutionized our knowledge of disease-associated gene expression profiles (Yofe et al., 2020) and exhibit advantages in elucidating the complexity and heterogeneity of tumors, which are composed by cancer cells, immune cells, and stromal cells (Bruni et al., 2020). Considering the complexity of the interplay between the autophagy and tumor microenvironment, high-dimensional methods need to be applied to construct the prognostic model. Therefore, to comprehensively identify the predictive biomarkers for early diagnosis of GC, we aimed to elucidate the significance of autophagy-related genes in the prognosis and clinical management of GC, both utilizing the analysis of bulk RNA sequencing and scRNA-seq.

Here, we identified an autophagy-related risk model through the integrative analysis of bulk RNA sequencing and scRNA-seq. The capability of this model in predicting the prognosis of patients with GC was validated. The potential application of the autophagy-associated risk model in facilitating the identification of the predictive biomarkers in immunotherapy response was also explored.

## Materials and Methods

### Data Acquisition

The expression matrix data from GSE62254, GSE15459, GSE134520, GSE146027, and GSE91061 datasets were directly downloaded from Gene Expression Omnibus (http://www.ncbi.nlm.nih.gov/geo/). The scRNA-seq count matrix has been described by Zhang et al. (2019). Additionally, the training cohort, which contained 255 GC samples, was obtained from TCGA (https://genome-cancer.ucsc.edu/).

### Identification and Assessment of Autophagy-Associated Gene Expression Data

The autophagy-associated expression data used in our study are available on the Human Autophagy Database (HADb, http://autophagy.lu/clustering/index.html) and GSEA database (https://www.gsea-msigdb.org/gsea/index.jsp). The LASSO Cox regression model analysis was performed utilizing the “glmnet” package of R software, and random forest regression was analyzed utilizing the “randomForestSRC” package of R software (version 3.6.1). As described previously (Tian et al., 2017), the penalized Cox regression model with LASSO penalty was applied for the shrinkage and variable selection simultaneously. The optimal values of the penalty parameter lambda were evaluated through 10-times cross-validations. A list of prognostic genes with associated coefficients was screened out according to the optimal lambda value. The risk score for each patient was calculated according to the expression level of each prognostic gene and its related coefficient. The patients in each dataset were classified into a low-risk group and a high-risk group based on the median risk score. The Kaplan–Meier estimator and the log-rank test were performed to evaluate the survival differences between the two groups.

### Analysis of Single-Cell RNA Sequencing Data

All the analyses of single-cell RNA sequencing were performed with SCANPY (Wolf et al., 2018) (version 1.5.1) in Python (version 3.6). The principal component analysis (PCA) and t-distributed stochastic neighbor embedding (t-SNE) methods were used for the dimension reduction. Clusters were annotated according to the marker genes. The pathway activity scores, which were evaluated by the mean expressions of the pathway gene set downloaded from the Molecular Signatures database (MsigDB; https://www.gsea-msigdb.org/gsea/msigdb/index.jsp), were computed using “sc.tl.score_genes ()” function by default parameters.

### Construction of the Nomogram

The nomogram and calibration plots were performed utilizing the “rms” package of R software (version 3.6.1). The concordance index, which indicated the level of consistency between the actual observed outcome frequencies and predicted probabilities, was used to evaluate the predictive accuracy of a nomogram. Cross-validation was applied for evaluating the model overfitting. A bootstrap resampling method was utilized to assess the confidence interval (CI) of concordance indexes. The performance of the nomogram was visualized by a calibration plot.

### Statistical Analysis

All the analyses were performed using R software (version 3.6.1). Multivariable Cox regression was utilized to test whether the risk score was independent of other known risk factors. The receiver operating characteristic (ROC) analysis was conducted to evaluate the sensitivity and specificity of the survival prediction according to the risk score, AJCC stage, and the combined model. A *p* value less than 0.05 was defined as the significant difference for all the statistical analyses.

## Results

### Autophagy Closely Relating to the Progression of Gastric Cancer at Single-Cell Resolution

**FIGURE 1:**
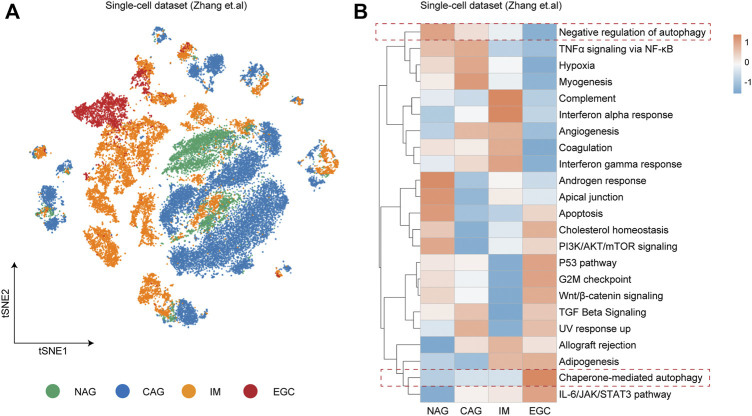
Autophagy closely relating to the progression of gastric cancer at single-cell resolution. **(A)** t-distributed stochastic neighbor embedding (t-SNE) plot colored by different sample groups including non-atrophic gastritis (NAG), chronic atrophic gastritis (CAG), intestinal metaplasia (IM), and early gastric cancer (EGC). **(B)** Heatmap showing the expression-based pathway activities scored per cell within different sample groups as indicated in **(A)**.

To investigate the essential pathways involved in the progression of GC, we utilized the single-cell dataset from the study by Zhang et al. (2019), in which cells from non-atrophic gastritis (NAG), chronic atrophic gastritis (CAG), intestinal metaplasia (IM), and early gastric cancer (EGC) were captured by the single-cell RNA sequencing (Figure 1A). During the progression of GC by the NAG-CAG-IM-EGC sequence, the activity scores of pathways involved in cancer progression from the HALLMARK and Gene Ontology (GO) databases were calculated in each single cell within different sample groups. Intriguingly, we detected that the pathway score of “Chaperone-mediated autophagy” was gradually enhanced along the progression of GC by the NAG-CAG-IM-EGC sequence (Figure 1B). Oppositely, the pathway score of “negative regulation of autophagy” was consistently decreased during the progression of GC by the NAG-CAG-IM-EGC sequence (Figure 1B). Such observation indicated that the activation of autophagy might have the key connection with the progression of GC at single-cell resolution.

### Establishment and Validation of an Autophagy-Related Risk Score for Predicting the Prognosis of Gastric Cancer

To establish an autophagy-related risk model, we identified 567 autophagy-related genes derived from the Human Autophagy database (HADb) and the Molecular Signatures database (MSigDB). The LASSO Cox regression model and the random forest regression model were both applied to build the prognostic signature. Then, we identified an autophagy-related signature and two lists of probes with associated coefficients which were generated from the LASSO analysis and random forest regression analysis (Supplementary Table S1–S2 **)**. Considering the higher statistically significant value in predicting the prognosis of GC patients by the LASSO analysis compared with the random forest regression analysis, we chose the model generated from the LASSO analysis for further investigation (Supplementary Figure S1). The prognostic analysis of autophagy-related genes in GC patients from the TCGA cohort is detailed in Supplementary Table S3. Among the gene set of autophagy-related signature, 16 genes had positive coefficients, including *NEK7*, *MAP1LC3C*, *FBXL2*, *TP63*, *SNCA*, *CDH6*, *SMURF1*, *LETM2*, *IFNA4*, *IFNA17*, *IFNA2*, *GAL3ST3*, *GABARAPL2*, *ITGA3*, *HGS*, and *USP9Y*. The coefficients for the other six genes (*ATG4C*, *CD46*, *ATG16L1*, *UBQLN1*, *TSC1*, and *MTM1*) were negative.

**FIGURE 2:**
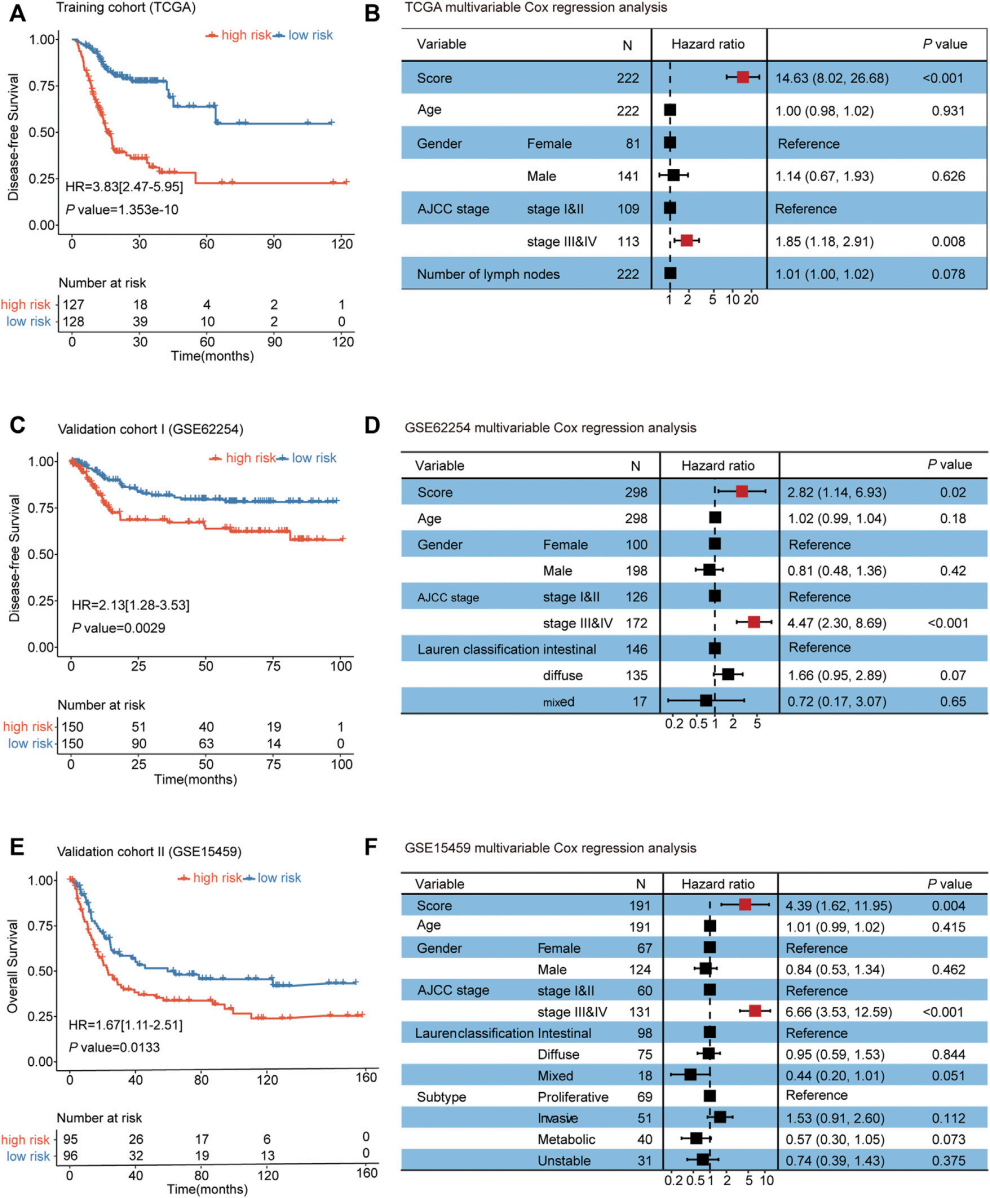
Establishment and validation of an autophagy-related risk score for predicting the prognosis of gastric cancer. **(A)** Kaplan–Meier curves for TCGA patients (*n* = 255). **(B)** Multivariable Cox regression analysis of the risk score, age, gender, AJCC stage, and number of lymph nodes on TCGA. **(C)** Kaplan–Meier curves for GSE62254 patients (*n* = 300). **(D)** Multivariable Cox regression analyses of the risk score, age, gender, AJCC stage, and Lauren classification on GSE62254 datasets. **(E)** Kaplan–Meier curves for GSE15459 patients (*n* = 191). **(F)** Multivariable Cox regression analyses of the risk score, age, gender, AJCC stage, Lauren classification, and subtype on GSE15459 datasets. The differences between the two curves were determined by the two-sided log-rank test. The squares on the transverse lines in the forest plot represent the hazard ratio (HR), and the transverse lines represent 95% CI. Risk score, age, and number of lymph nodes are continuous variables; gender, AJCC stage, Lauren classification, and subtype are discontinuous variables.

Next, we validated the prognosis value of the autophagy-related risk score in GC. In the survival analysis, the autophagy-related signature risk score for each patient was calculated in TCGA, and a dichotomous score was adopted. Patients with GC from the TCGA database were divided into a low-risk group (*n* = 128) and a high-risk group (*n* = 127) according to the median risk score. A worse disease-free survival (DFS) was demonstrated in the high-risk group than in the low-risk group (HR = 3.83, 95% CI 2.47–5.95, *p* < 0.001) (Figure 2A, Supplementary Table S4). To investigate whether the prognostic value of the autophagy-related risk score was independent of other clinical factors associated with the progression of GC, the multivariable Cox regression analysis was performed using the risk score, age, gender, tumor stage, and number of lymph nodes as covariates. We demonstrated that the autophagy-related risk score was significantly related to a worse prognosis as a continuous variable (*p* < 0.001) (Figure 2B, Supplementary Table S5).

The efficacy of the autophagy-related risk score for predicting the prognosis of patients with GC was further validated in two independent datasets (GSE62254 and GSE15459). Consistently, patients from the high-risk group in the validation cohorts Ⅰ and Ⅱ exhibited the significantly worse prognosis than patients from the low-risk group (Figures 2C, E, and Supplementary Table S6–S7). The multivariable Cox regression analyses also showed that the association of the autophagy-related risk score with the prognosis of GC was statistically significant as a continuous variable in the two validation cohorts (Figures 2D, F, and Supplementary Table S8–S9). Our findings above all suggested that the autophagy-related risk score model exhibited the unbiased efficacy for predicting the prognosis of gastric cancer.

### The Clinical Value of the Autophagy-Related Risk Model in Gastric Cancer

**FIGURE 3:**
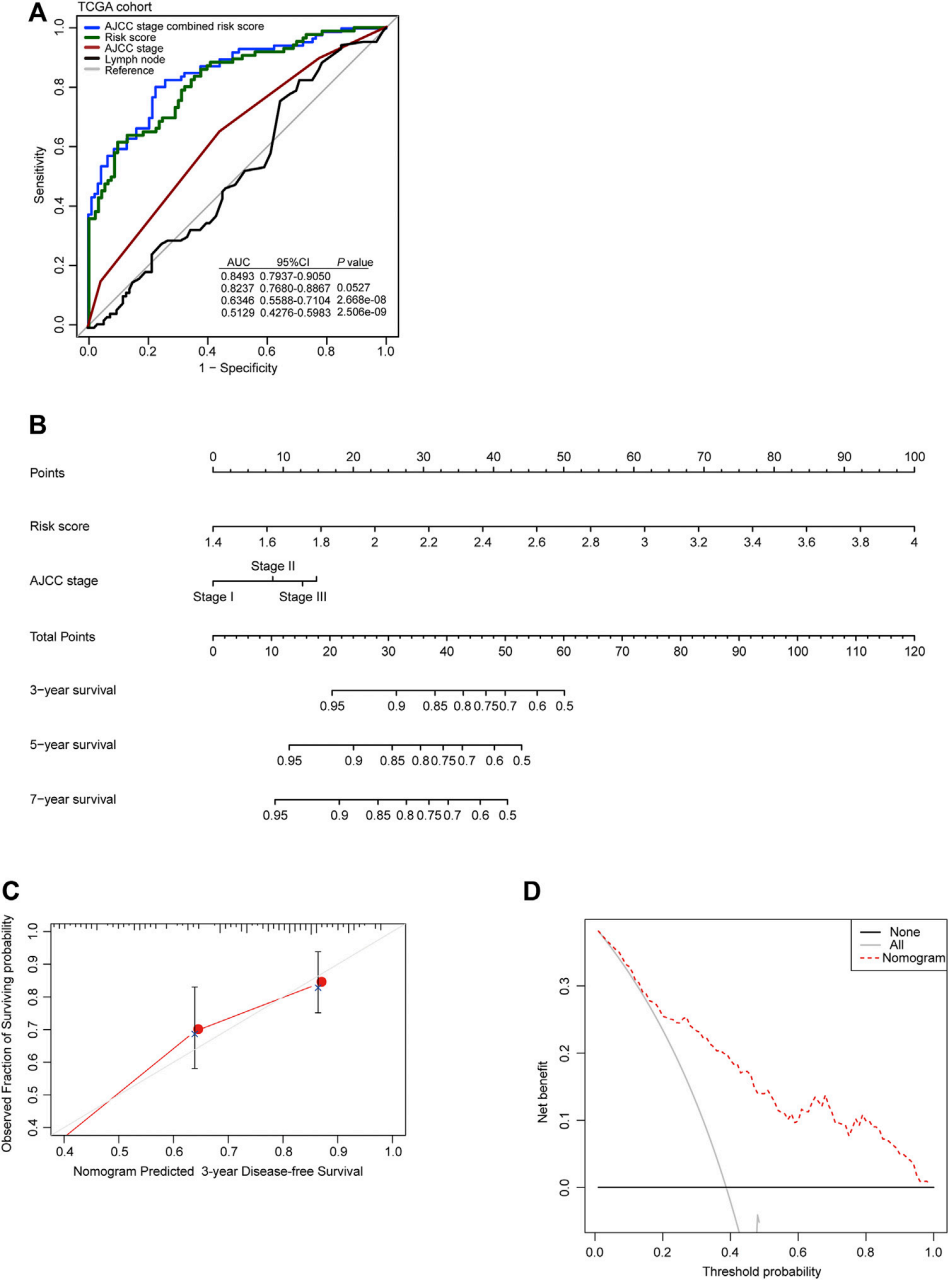
Clinical value of the autophagy-related risk score model in gastric cancer. **(A)** Receiver operating characteristic (ROC) analysis of the sensitivity and specificity of the recurrence prediction by the risk score, AJCC stage, and the number of lymph nodes in TCGA cohort. *p*-values were from the comparisons of the area under the ROC curve (AUC) of risk score combined with AJCC stage versus risk score and AJCC stage separately. **(B)** The nomogram for predicting the proportion of patients with disease-free survival. **(C)** The calibration plots for predicting disease-free survival. Nomogram-predicted probability of recurrence is plotted on the *x*-axis; actual recurrence is plotted on the *y*-axis. The solid line represents our nomogram and the vertical bars represent 95% CIs. **(D)** Decision curve analysis (DCA) for assessment of the clinical utility of the nomogram. The *x*-axis represents the percentage of threshold probability, and the *y*-axis represents the net benefit.

To further evaluate the clinical value of the autophagy-related risk score in the management of patients with GC, the ROC analysis was performed to demonstrate the sensitivity and specificity of survival prediction in patients with GC. The area under the ROC curve (AUC) was evaluated and compared between the autophagy-related signature score and the AJCC cancer staging system. Our observation indicated that the autophagy-related risk model possessed the strongest predictive power compared to the AJCC cancer staging system and the number of lymph nodes for the prognostic evaluation of GC patients from the TCGA database (0.8237, 95% CI 0.7680–0.8867). The efficacy of diagnosis using the AJCC stage was significantly enhanced when combined with the autophagy-related risk score (0.8493 vs 0.6346, 95% CI 0.7937–0.9050 vs 0.5588–0.7104, *p* < 0.001) (Figure 3A).

Then, to develop a practical model for clinicians to predict the probability of 3- and 5-year DFS in GC, a nomogram was constructed, which integrated the autophagy-related risk score and the AJCC stage (Figure 3B). The line-segment in the calibration plot was very close to the 45° line, which represented the best prediction utilizing the nomogram (Figure 3C). The decision curve indicated that utilizing the nomogram for the prediction of recurrent probability added more benefit than the treat-all-patients scheme or the treat-none scheme when the threshold probability of DFS of a patient was more than 15% (Figure 3D). Our observations above all suggested the reliable predictive ability of the autophagy-related risk model in clinical management of patients with GC.

### Alterations in the Feature of T Cell Between Different Risk Groups Predicted by an Autophagy-Related Risk Model

**FIGURE 4:**
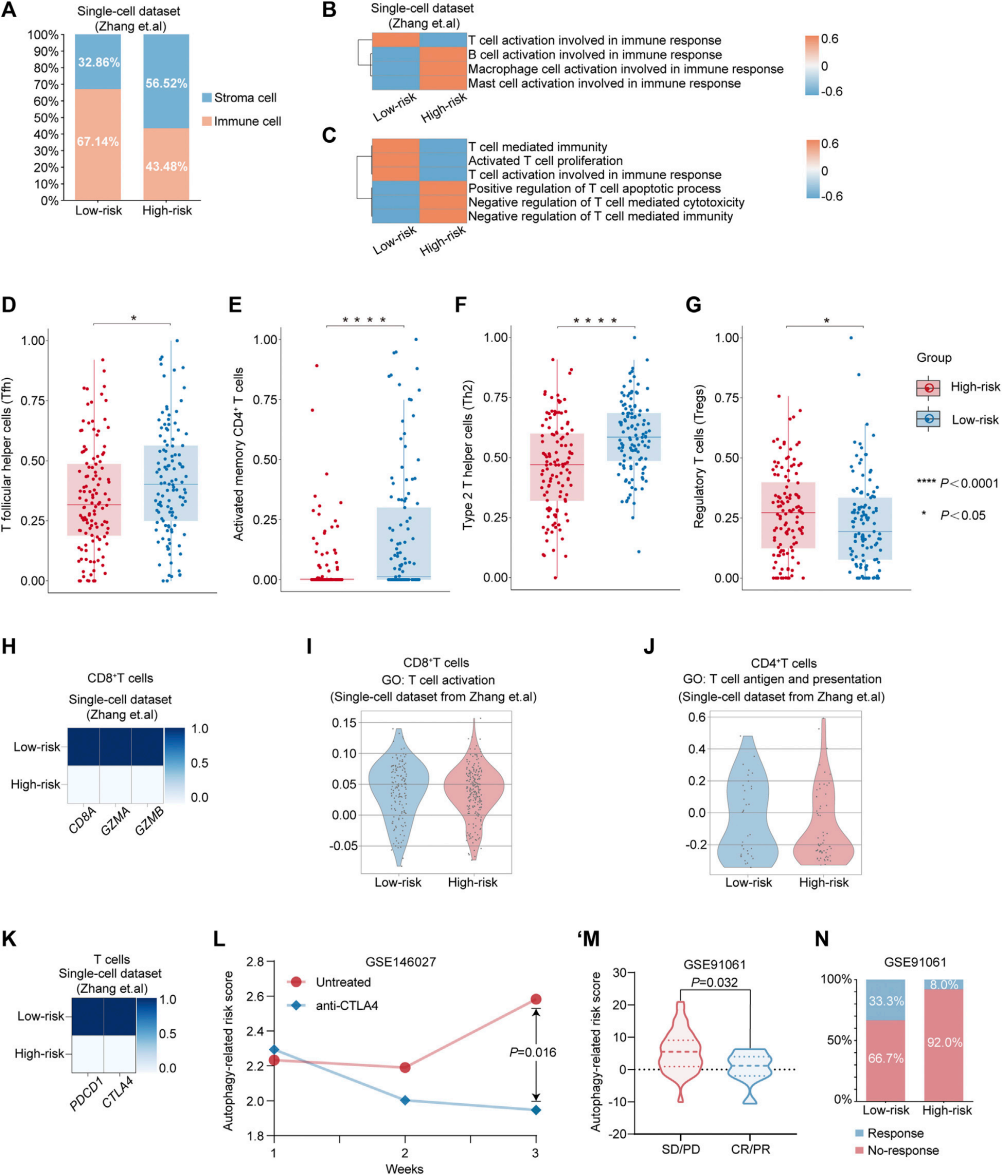
Alterations in the feature of T cell between different risk-groups predicted by autophagy-related risk model. **(A)** Percentages of immune cell and stromal cells in different risk groups in TME. **(B–C)** Heatmap showing the expression-based pathway activities scored per cell between the low-risk group and the high-risk group. **(D–G)** The different compositions of T follicular helper cells (Tfh) **(D)**, activated memory CD4^+^ T cells **(E)**, type 2 T helper cells (Th2) **(F)** and regulatory T cells (Tregs) **(G)** between low-risk patients and high-risk patients from TCGA dataset. *p* value was calculated by nonparametric Mann–Whitney test. *p value < 0.05. ****p value < 0.0001. **(H)** Matrix plot showing the mean expression of *CD8A*, *GZMA*, and *GZMB* between the low-risk group and the high-risk group. **(I)** Violin plot showing the distribution of activity score of “T cell activation” from GO database in CD8^+^ T cells from the low-risk group and the high-risk group, respectively. **(J)** Violin plot showing the distribution of the activity score of “T cell antigen and presentation” from GO database in CD4^+^ T cells from the low-risk group and the high-risk group, respectively. **(K)** Matrix plot showing the mean expression of *PDCD1* and *CTLA4* in T cells from the low-risk group and the high-risk group, respectively. **(L)** The alterations of autophagy-related risk score in untreated or anti-CTLA-4 mAbs groups in the GC mouse model at week 1, 2, and 3 from Nagaoka et al.’s dataset. *p* value was calculated by nonparametric Mann–Whitney test. **(M)** Violin plot showing the expression of autophagy-related risk scores in patients with PD/SD or PR/CR from GSE91061. **(N)** Differential response rate of immune checkpoint blockade therapy in the low-risk group and the high-risk group, respectively. CR, complete response; PR, partial response; SD, stable disease; and PD, progressive disease.

Considering the complexity of TME during the progression of cancer, we first calculated the proportions of immune cells and stromal cells in the high-risk and low-risk groups in single-cell dataset from Zhang et al., respectively. Compared with the high-risk group (43.48%), the TME of the low-risk group was infiltrated with a higher percentage of immune cells (67.14%) (Figure 4A). Additionally, by evaluating the scores associated with activation involved in immune response among different immune subsets, including T cells, B cells, macrophages, and mast cells, we observed that only the score of T-cell activation involved in immune response was enriched in the low-risk group (Figure 4B). Thus, given that T cells also play the key role in modulating the progression of GC and the clinical response of immunotherapy (Kwon et al., 2021; Poffenberger et al., 2018), we query whether the better prognosis in GC patients from the low-risk group was related to the activation of T-cell immune function. Intriguingly, we detected that pathways associated with immune effector function, including “T-cell activation involved in immune response,” “Activated T-cell proliferation,” and “T-cell–mediated immunity,” were enriched in the low-risk group, while they were decreased in the high-risk group in the single-cell dataset from the study by Zhang et al. (Figure 4C). Additionally, pathways associated with the negative regulation of anti-tumor immune, such as “Negative regulation of T-cell–mediated cytotoxicity” and “Negative regulation of T-cell–mediated immunity,” were enriched in the high-risk group (Figure 4C). Then, to investigate the alterations in the composition of T-cell subsets, we divided patients from TCGA into the low-risk group and the high-risk group according to the autophagy-related risk model. The CIBERSORT algorithm (Newman et al., 2015) was utilized to evaluate the average proportion of each T-cell subset. We observed that the compositions of T follicular helper cells (Tfh), activated memory CD4^+^ T cells, and type 2 T helper cells (Th2) were significantly decreased in the high-risk group. Yet, the proportion of regulatory T cells (Tregs), which had been implicated in the cancer progression, was significantly elevated in the high-risk group (Figures 4D–G).

Furthermore, we re-clustered T cells from the single-cell dataset into CD8^+^ T cells and CD4^+^ T cells for further investigation (Supplementary Table S10–S11). Intriguingly, we observed that the expressions of genes associated with the CD8^+^ T-cell–mediated cytotoxicity, including *CD8A*, *GZMA*, and *GZMB*, were markedly decreased in the high-risk group (Figure 4H). Additionally, the distribution of the activity score of “T-cell activation” was decreased in CD8^+^ T cells from the high-risk group (Figure 4I) and the activity score of “T cell antigen and presentation” was also exhibiting the decreased tendency in CD4^+^ T cells from the high-risk group compared with the low-risk group (Figure 4J), which indicated the poor prognosis in high-risk GC patients might be associated with the dysregulation of T cell-function. As immunotherapy has opened a new era of therapy in GC (Janjigian et al., 2021; Chao et al., 2021; Salas-Benito et al., 2021), we further investigated whether the poor prognosis of the high-risk patients was related to the invalid response for immunotherapy. Intriguingly, we detected that the expressions of the two essential immune checkpoint gene markers: *PDCD1* (encoding PD-1) and *CTLA4* (encoding CTLA4), were markedly increased in the low-risk group, while they were decreased in the high-risk group (Figure 4K). To verify the efficacy of the autophagy-related risk score in predicting the response of immunotherapy, we utilized two public datasets for evaluation. In the GC mouse model with the treatment of anti–CTLA-4 mAbs, the autophagy-related risk score was significantly decreased at week 3 compared with the untreated group from the study by Nagaoka et al. (Figure 4L) (Nagaoka et al., 2020). Moreover, in patients cohort treated with checkpoint blockade therapy (Riaz et al., 2017), we revealed that the autophagy-related risk score was significantly elevated among patient with stable disease [SD] or progressive disease [PD] by a comparison with patients who had complete response [CR] or partial response [PR] (Figure 4M). Patients in the low-risk group demonstrated higher response efficacy of immunotherapy (33.3%) than patients in the high-risk group (8.0%) (Figure 4N). Such alterations between the two risk groups predicted by the autophagy-related risk model indicated that the poor prognosis in patients from the high-risk group might account for the emergence of dysfunctional characteristics in T cells, and patients with a low-risk score exhibited the potential in response to immunotherapy.

## Disscussion

A large body of evidence has demonstrated that autophagy may be implicated in numerous aspects of cancer progression, prognosis, and treatment response (Cao et al., 2019). However, the traditional method singly derived from bulk RNA sequencing still exhibits several limitations in elucidating the complexity of oncology. In current years, single-cell RNA sequencing has broken the conventional thinking in the mechanism of cancer progression (Keller and Pantel 2019; Yofe et al., 2020). Therefore, through the combined analysis of bulk RNA sequencing and single-cell RNA sequencing, the advantages in investigating and validating the value of prognostic model in gastric cancer are shown.

In the study, an autophagy-related risk model was investigated according to the machine learning–based computational method and was validated both in the bulk RNA sequencing dataset and single-cell RNA sequencing dataset. Our results demonstrated that this classifier could effectively classify GC patients into high-risk and low-risk groups, which had the ability to distinguish the patients with significantly different DFS. Even when the conventional clinical factors were adjusted (Shen et al., 2013), the risk model remained an independent prognostic factor, which was in a position to distinguish worse vs relieved prognostic outcomes within patients with GC from different datasets. Such a prognostic value of this risk model could be identified in another two independent datasets, suggesting the reproducibility and practicality of this risk model for the prognostic prediction in patients with GC. Particularly, recent studies have demonstrated that genes involved in our risk model, such as Smad ubiquitin regulatory factor 1 (*SMURF1*) and NIMA-related kinase 7 (*NEK7*), played the essential roles in promoting the progression of GC (Ji et al., 2018; Jiang et al., 2019; Li et al., 2021). Experimental evidence indicated that SMURF1 exerted a pro-tumorigenic role by regulating the downstream pathways and influenced the epigenetic mechanism (Ji et al., 2018; Jiang et al., 2019). NEK7 was indicated to not only promote the progression of GC but also closely associate with tumor immune infiltration (Li et al., 2021). Such evidence is consistent with our finding that our risk model may be the potentially prognostic indication in the progression of patients with GC. Although there had been several published studies on autophagy-related risk models, these models were all constructed and estimated according to the RNA sequencing generated from the bulk level and singly focused on the tumor cells. The risk model identified in our research was not only validated in cohort at bulk level but also estimated in samples at the single-cell level. In addition to the prognostic value in GC patients, the model could also be complementary with and add information to the predictive biomarkers of immunotherapy response in patients with GC.

Advances in the knowledge of immune checkpoint inhibitors have uncovered a new era of cancer immunotherapy (Janjigian et al., 2021; Chao et al., 2021; Salas-Benito et al., 2021). The involvement of autophagy in the differentiation, activation, and apoptosis of tumor-infiltrating immune cells has been demonstrated in several studies (Clarke and Simon 2019; Xia et al., 2021). The effector function of T cells and tumor immune response could be influenced and shaped by autophagy-associated pathways (Yamamoto et al., 2020; Xia et al., 2021). Yet, only a limited proportion of autophagy-associated biomarkers were investigated, indicating the urgent demands for the investigation of predictive autophagy-associated models. PD1 and CTLA4 were well-known predictive biomarkers for immunotherapy response (Havel et al., 2019). A recent study has revealed that pharmacological modulation of autophagy might affect the therapeutic efficacy of PD-L1 blockade in GC (Wang et al., 2019). Interestingly, a growing body of studies identified that CD46, a variable involved in our risk model with a negative coefficient, exerted an essential role in modulating the effector function of T cells (Arbore et al., 2018; Liszewski and Atkinson 2021). In consistency with previous investigation that immunomodulatory interactions between the autophagy and cancer (Jacquin and Apetoh 2018; Xia et al., 2021), we also identified that dysfunctional characteristics enriched in the autophagy-associated high-risk group and the low-risk group exhibited high expression of immune checkpoint gene markers and genes associated with the CD8^+^ T-cell–mediated cytotoxicity than those in the high risk group. Such observations, in combination with previous findings, indicated the potential association between the autophagy-associated risk score model and effector function of immune cells.

Taken together, our findings revealed that the autophagy-related risk model could not only be a useful tool for prognostic evaluation but also be a complementary with and add information to the predictive biomarkers of immunotherapy response in patients with GC.

## Data Availability

The datasets presented in this study can be found in online repositories. The names of the repository/repositories and accession number(s) can be found in the article/Supplementary Material.
