# Supplementary figures and images for: Prognostic Autophagy-Related Model Revealed by Integrating Single-Cell RNA Sequencing Data and Bulk Gene Profiles in Gastric Cancer
Source: Front Cell Dev Biol. 2022 Jan 10;9:729485. doi: 10.3389/fcell.2021.729485 (PMC8785981; doi:10.3389/fcell.2021.729485)

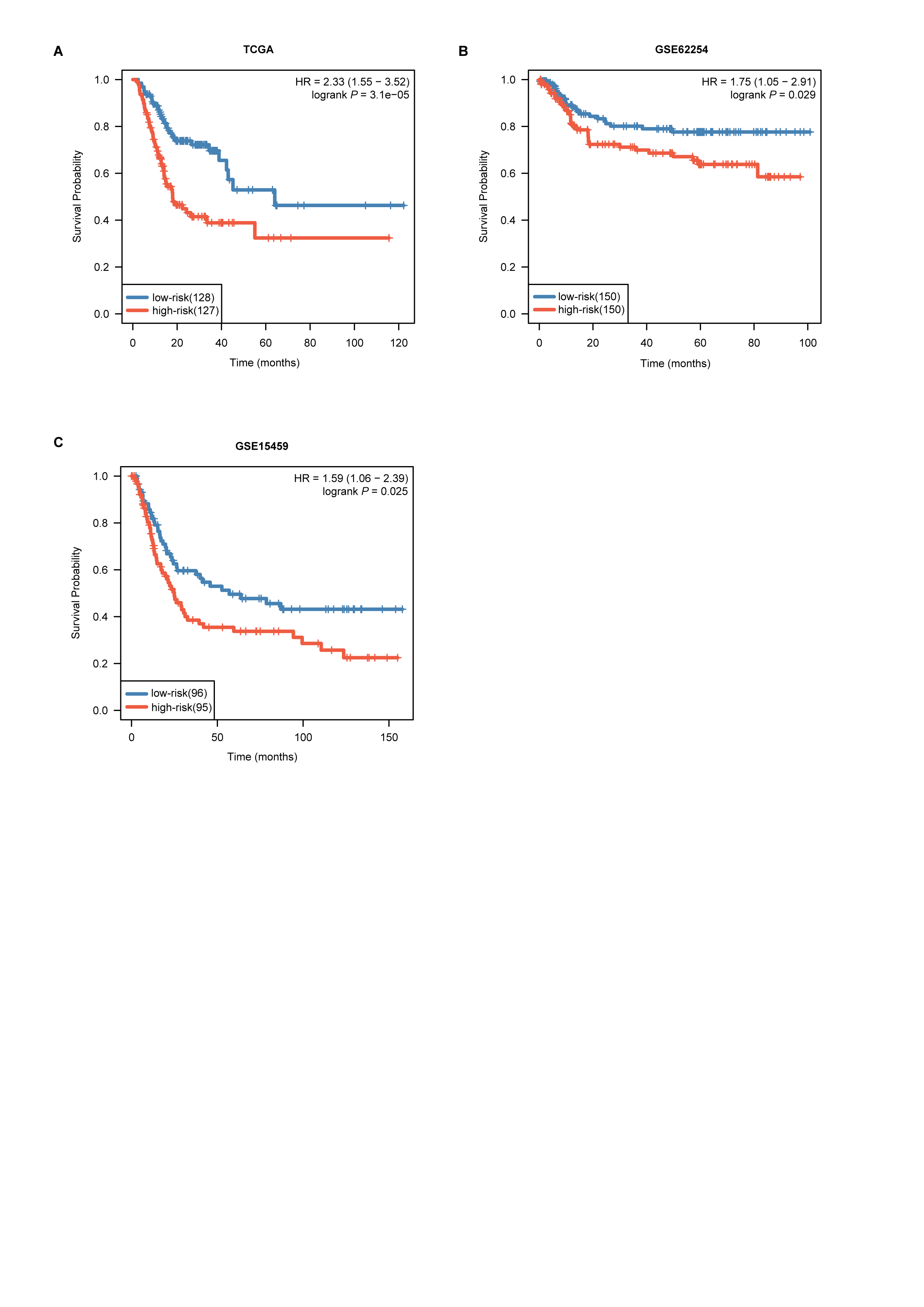

Supplement: Supplementary file 4 [file Image1.TIF]
